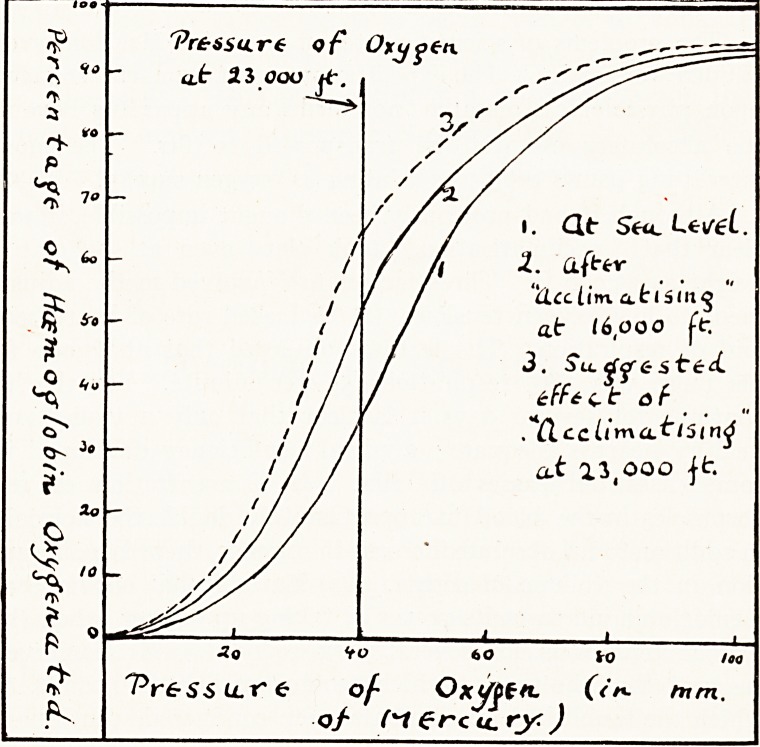# Meetings of Societies

**Published:** 1923-07

**Authors:** 


### Meetings of Societies.

The Meeting of the Society, held on May 16th in the Lecture Theatre of the Bristol Museum and Art Gallery, was an event of exceptional interest ; Mr. T. Howard Somervell, F.R.C.S., one of the members of the recent expedition to Mount Everest, gave an address on the subject of " The Problems of Living at High Altitudes." There were present : the President, Dr. J. A. Nixon, C.M.G., in the Chair ; the Pro-Chancellor of the University, Mr. G. A. Wills ; the Sheriff of Bristol, Mr. Horace Walker, D.L., J.P.; the Master of the Society of Merchant Venturers, Mr. Gerald Beloe ; and about 300 members and their guests. The address was illustrated by lantern slides from the author's camera and by parts of the official film, showing the actual ascent of the mountain. The country through which the expedition approached the mountain was shown, with interesting accounts of the Tibetan manners and customs. The lecturer then described the actual ascents made by three parties, of which he took part in two. The film enabled one to imagine the vast physical obstacles that were encountered.

The actual attempts on the mountain were made from an advanced base camp at a height of 23,000 ft. The expeditions from this point calculated to be away for four days at a time. The first attempt, made without oxygen, reached an altitude of 27,000 ft.; the second, with oxygen, 27,250 ft.; the third was, as all remember, overwhelmed in an avalanche, with seven deaths. This disaster and the bad weather which caused it brought the expedition to a close.

The problems of scientific interest to which Mr. Somervell alluded were many. The expedition was not primarily engaged upon physiological research, nor could any apparatus beyond the absolutely essential be transported so far. The most interesting points are those relating to oxygen supply.

Although it had previously been thought impossible, it was clear that "acclimatisation" took place even at so great a height as 23,000 ft. Three factors are involved in the adjustment to low oxygen tension: (1) Increased rate of heart beat and of respiration. The lecturer observed that at 27,000 ft. his pulse was 180, respirations 55 per minute. For this to continue for several days it is clear that only a young and healthy heart is adequate; a mitral insufficiency developed in some cases but passes off later. (2) Concentration of red corpuscles in the blood to approximately double the normal; in addition to an absolute increase in number, there is a diminution in the volume of serum. (3) Barcroft has shown that hæmoglobin increases its power of taking up oxygen when the oxygen tension is for several days reduced. Mr. Somervell suggests that the process which occurs in rising to 16,000 ft., at which the published experiments were conducted, continues; and points out that if so nearly twice the "sea level" oxygenation will be found. In the sketch diagram (not to scale) the dotted line represents the suggested curve acclimatised at 23,000 ft.; a percentage of 65 oxygenation instead of the 35 per cent. at "sea level adjustment" is shown on next page. The combined effect of these processes was very noticeable after several days' living and climbing at 23,000 ft.

During the early part of the ascent to the advanced base camp mountain sickness was noticed by everyone at 17,000 ft., but it soon passed off. At the higher camp appetites failed, but in many cases they returned as acclimatisation occurred. Administration of oxygen was also found to restore the appetite. Anorexia appeared to be a definite effect of oxygen starvation. This symptom proved serious in one or two of the older members of the party; they did not "acclimatise," and partial starvation increased their troubles at the higher altitudes.

In the second attempt oxygen in cylinders sufficient for eight hours was carried; the apparatus, however, weighed thirty-four pounds, and this, in Mr. Somervell's opinion, more than counterbalanced its advantages. The fact that eight Nepali bearers were required to carry the apparatus to the advanced camp (from the advanced base) was a serious factor. Mr. Somervell felt convinced that better results will be obtained by men who acclimatise at high altitudes and then ascend without the apparatus, as by these tactics a larger number of attempts can be made in one season.

Cold was severe; twelve thicknesses of clothing were worn, and more would have been better; only one member suffered severely from frost bite. The cold itself recorded was never below $-20°$ F, but it was rendered more severe in effect by the high wind. Atmospheric conditions, however, are of the utmost importance. Snow or a high wind render an ascent out of the question, and these are apt to prevail on two days out of three. The monsoon makes the snow unsafe for climbing; a recent fall of soft snow resulted in the fatal avalanche which terminated the third attempt. Thirst was a severe enemy; water could only be obtained by melting the snow, and therefore depended on fuel; the allowance of drinking water was very limited during the ascent. One member of the first party became seriously ill from thirst and could not continue the climb. The party were thirty-six hours without water, and were unable, in consequence, to eat.

The mental effects of oxygen-want were interesting. All the climbers suffered from bad temper. A certain irresponsibility was observed. Mr. Somervell did not suffer any disappointment at having to abandon the first climb. The second party took a camera up and forgot to use it.

The lecturer concluded by expressing a hope that another British expedition might be equipped to go out in 1924. This was solely a question of funds; £14,000 was needed to make another attempt. If the attempt were delayed possibly some other nation would "get there first."

Dr. P. Watson-Williams moved a vote of thanks to the lecturer for his admirable account, and expressed the pleasure it and the unique films had given. He was convinced that Bristol would lead the way in equipping next year's expedition. In this conviction Mr. Walker, the Sheriff, and Mr. Beloe, Master of the Merchant Venturers, joined. Mr. T. Carwardine seconded the vote, which was carried by acclamation.

## Figures and Tables

**Figure f1:**